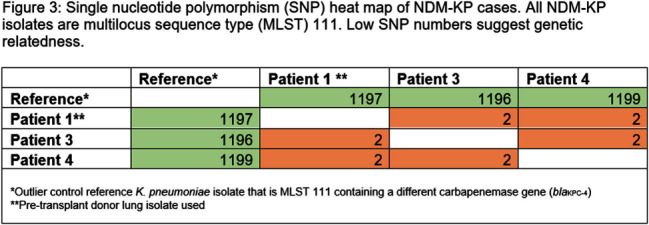# Investigation of a Donor-derived Carbapenamase-producing Carbapenem-resistant Enterobacterales Hospital Outbreak

**DOI:** 10.1017/ash.2024.288

**Published:** 2024-09-16

**Authors:** Alice Lehman, Ginette Dobbins, Jesse Sutherland, Megan Krieglmeier, M Health Fairview, Jessica Kanelfitz, Terra Menier, Jennifer Dale, Susan Kline, Patricia Ferrieri, Alison Galdys

**Affiliations:** University of Minnesota; Minnesota Department of Health; M Health Fairview; University of Minnesota Medical Center

## Abstract

Carbapenamase-producing carbapenem-resistant Enterobacterales (CP-CRE) is an urgent public health threat for healthcare facilities. Solid organ transplant (SOT) recipients carry an increased risk for CRE infection and colonization due to prolonged exposures to antimicrobials, healthcare facilities and immunosuppression. CRE infection in SOT patients is associated with an increase in morbidity and mortality. Here, we describe a hospital outbreak investigation of three cases of New Delhi metallo-beta-lactamase (NDM) - CRE that led to novel findings with implications for further interdisciplinary investigations. An NDM-CRE infection in a critically-ill patient was identified during passive surveillance and prompted an investigation. Previous CP-CRE passive surveillance cases were reviewed. Rectal screening was performed for potentially exposed patients. 403 rectal swabs were tested for carbapenemase genes in active surveillance. Patients identified to have a new NDM-CRE isolate on active or passive surveillance were considered cases and underwent in-depth chart review including possible patient-to-patient exposures, hospital locations, procedures, devices, and consultations. NDM-CRE isolates were sent to the Minnesota Department of Health (MDH) for whole genome sequencing (WGS) to assess relatedness. Five NDM-CRE cases were identified, with all isolates harboring blaNDM including three NDM-Klebsiella pneumoniae (NDM-KP) cases (Figure 1). The first NDM-KP case, patient 1, developed mediastinal infection following lung transplantation. Review of United Network for Organ Sharing revealed that respiratory specimens from patient 1’s donor grew NDM-KP and a bronchial wash at the time of transplant yielded NDM-KP. The second NDM-KP case (patient 3) developed ventilator-associated pneumonia and was found to have used sequentially the same ventilator as patient 1. The third NDM-KP case (patient 4) was detected via rectal swab in active surveillance and shared wound care personnel in common with patients 1 and 3 (Figure 2). WGS demonstrated two single nucleotide polymorphisms (SNP) among all three isolates, strongly suggesting relatedness (Figure 3). Best practices for infection prevention were reviewed with wound care personnel. To date, no further NDM-KP isolates have been identified. Investigation was facilitated by in-depth chart review and WGS via the Central Region Antimicrobial Resistance Laboratory Network at MDH. Detection of the NDM-KP from a lung donor specimen appears genetically linked to clinical isolates in other patients, raising the possibility of a donor-derived hospital outbreak. This investigation is the first to describe a donor-derived NDM outbreak in a healthcare facility. Communication between organ procurement agencies, transplant centers, and infection prevention must be optimized to prevent CRE-associated morbidity in SOT receipts and CRE hospital outbreaks.

**Figure:**
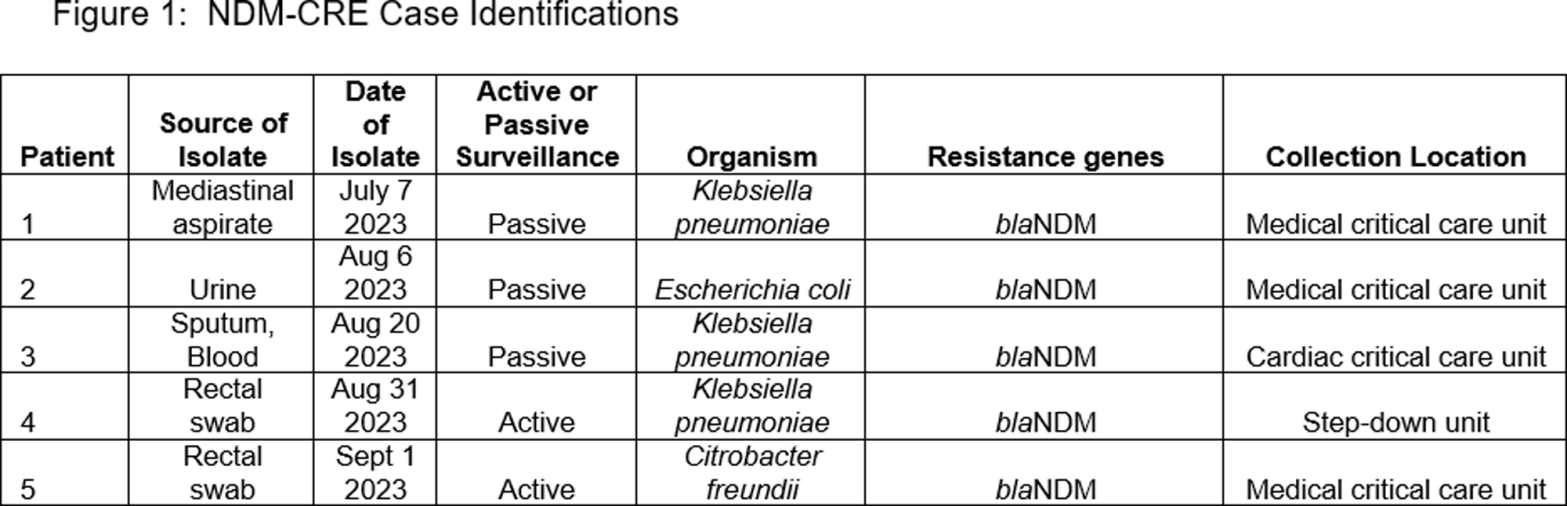

**Figure:**
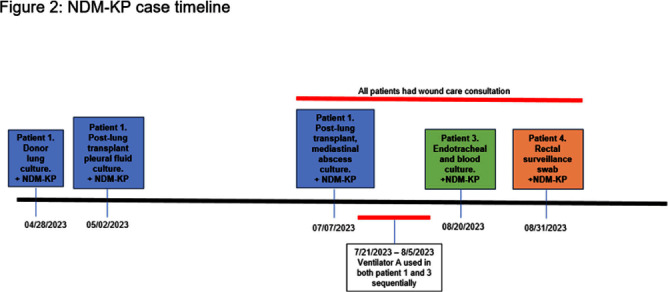

**Figure:**